# Keeping Our Eyes on Older Adults: Training CHWs in Geriatric Care

**DOI:** 10.1093/geroni/igaf122.3615

**Published:** 2025-12-31

**Authors:** Kevin Espinoza, Joycelyn Lawrence, Cherell Cottrell-Daniels, Katherine Chung-Bridges, Diego Shmuels, Deborah Gracia, Sweta Tewary

**Affiliations:** Health Choice Network, Inc., Miami, Florida, United States; Jessie Trice Community Health System, Miami, Florida, United States; Health Choice Network, Miami, Florida, United States; Health Choice Network, Miami, Florida, United States; Borinquen Healthcare Center, Inc., Miami-Dade, Florida, United States; Borinquen Healthcare Center, Inc., Miami-Dade, Florida, United States; Jessie Trice Community Health Center, Miami, Florida, United States

## Abstract

**Background:**

The growing aging population in the U.S. highlights the urgent need for specialized geriatric care and trained professionals. Community Health Workers (CHWs) play a vital frontline role in supporting older adults, yet many lack formal geriatric training. The Geriatrics Workforce Enhancement Program (GWEP), in partnership with a local Area Health Education Center (AHEC), implemented a training based on the 4Ms framework—What Matters, Medication, Mentation, and Mobility—to address this gap and equip CHWs with evidence-based, patient-centered approaches.

**Methods:**

Twelve Community Health Workers (CHWs) from Jessie Trice Community Health System and Borinquen Medical Centers participated in a 30-hour geriatric-focused training in October 2024, focused on social determinants of health (SDOH) among older adults (aged ≥65 years). Pre- and post-exams assessed knowledge. Additionally, focus groups explored CHWs’ experiences, training relevance, and future needs. Eleven CHWs participated in two separate virtual focus groups, which were audio-recorded and transcribed. Representative quotations will be included in the presentation.

**Results:**

Participants showed statistically significant knowledge gains, as measured by a Wilcoxon signed-rank test (z = 3.91, p=.003). Focus group findings revealed seven key themes: (1) enhanced knowledge and confidence; (2) practical skill application to address complex needs like housing and behavioral health; (3) interdisciplinary-CHWs and Health Navigator collaboration; (4) supportive onboarding; (5) electronic health records (EHR) training; (6) regular refresher sessions; and (7) gaps in community resource accuracy.

**Conclusion:**

Structured, evidence-informed training significantly enhances CHW capacity in geriatric care and addressing SDOH. Sustained investment in CHW training is essential to build an age-friendly workforce.

